# Relationship between temperature and T2 in subcutaneous fat and bone marrow at 3T

**DOI:** 10.1186/2050-5736-3-S1-P89

**Published:** 2015-06-30

**Authors:** Eugene Ozhinsky, Misung Han, Serena Scott, Chris Diederich, Viola Rieke

**Affiliations:** 1 University of California at San Francisco, San Francisco, California, United States

## Background/introduction

MR-guided high-intensity focused ultrasound (HIFU) for treatment of uterine fibroids and painful bone metastases uses the proton resonant frequency shift (PRF) for temperature monitoring in water-based tissues. However, PRF fails to detect temperature changes in tissues with high lipid content, such as fat and bone marrow. Previous studies have shown a change in T2 of subcutaneous fat, red and yellow bone marrow during treatments with focused ultrasound. The lack of calibration data for 3T acquisitions, however, makes it difficult to convert T2 values into maps of tissue temperature.

In this study we investigated the dependence of T2 temperature on temperature in porcine adipose tissue and bovine yellow bone marrow at thermal equilibrium at 3T.

## Methods

**Figure 1:**
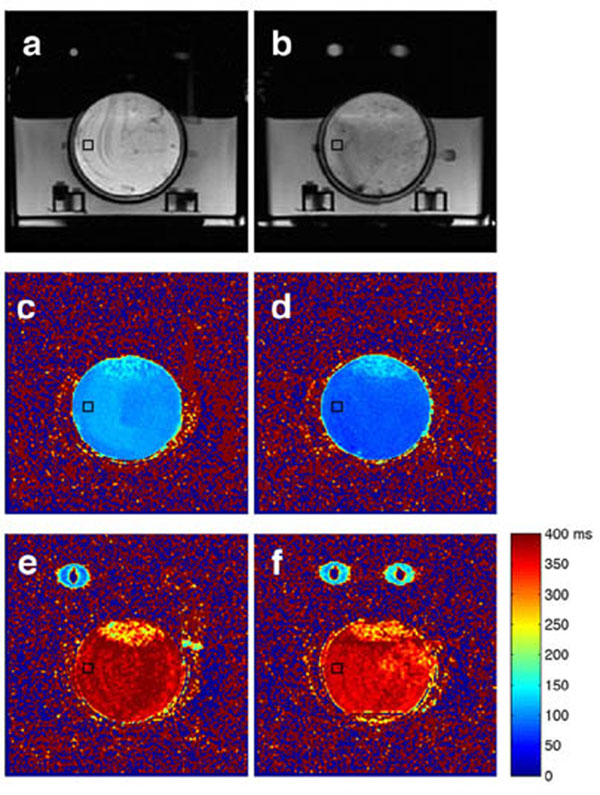
FSE images of petri dishes (no water suppression), containing porcine adipose tissue (a) and bovine yellow bone marrow (b); T2 maps of the tissues at 25°C (c, d); and at 70°C (e, f), acquired with water suppression.

Two petri dishes were filled with porcine adipose tissue and bovine yellow bone marrow (fig. 1a, 1b) and placed in a custom-built thermally insulated water bath that was held at a constant temperature by circulating water between scans. Temperature in the water was monitored with a fiber optic sensor. The time necessary for temperature equilibration between the T2 measurements was measured using fiber optic sensors, embedded in the samples. T2 was quantified in a 3T MRI scanner with a double-echo fast spin-echo sequence with and without water suppression (TE = 35/181 ms and 30/150 ms, TR = 1500 ms, ETL = 40, FOV = 12 cm, 128 x 128 matrix size, 8 mm slice thickness). Images were acquired during heating (25°, 35°, 45°, 55°, 65° and 70° C) and subsequent cooling (55°, 35° and 25°C) after reaching thermal equilibrium.

## Results and conclusions

**Figure 2:**
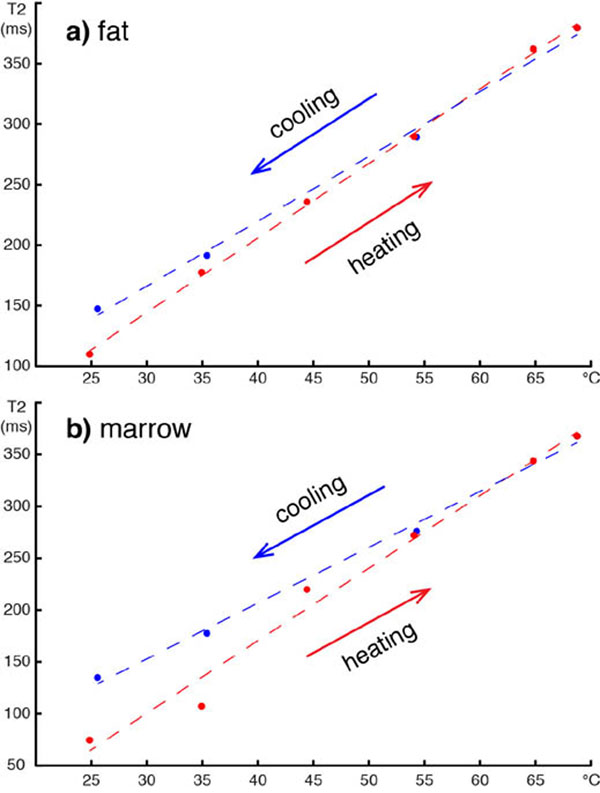


Figure 1(c-f) shows examples of T2 maps of fat and marrow at 25° and 70°C. The T2 values within a 10x10 pixel ROI (black square on fig. 1) *versus* the temperature of the water bath at equilibrium are plotted in Figure 2. The T2 values in the fat sample (fig. 2a) increased linearly with heating, but followed a different curve during cooling due to irreversible tissue changes around 45°C. The bone marrow sample exhibited a non-linear relationship between T2 and temperature during heating below 45°C (fig. 2b). As in the fat sample, the T2 values were higher and followed a more linear curve during cooling. Table 1 shows the linear regression coefficients of T2 *versus* temperature for the different acquisition parameters. There was approximately a 25% difference between the measurements with and without water suppression. This could be due to the contribution of water spins to the measured T2 in the non water-suppressed acquisitions and due to suppression of a portion of the fat spins in the water-suppressed acquisition. The difference in the T2/temperature coefficients between the two sets of echo-times was smaller. These results suggest that calibration of T2-based thermometry techniques should be done with the same parameters as those used for temperature monitoring during the treatment of patients. In conclusion, we have calibrated the temperature dependence of T2 in porcine subcutaneous fat and yellow bone marrow for temperatures between 25° and 70°C at 3T. This will allow for reliable and accurate monitoring of temperature in adipose tissues and yellow bone marrow during treatment of patients with MR-guided HIFU.

**Table 1:** Relationship between T2 and temperature (ms/°C) for porcine adipose tissue (fat) and bovine yellow bone marrow (marrow).

	Fat		Marrow	
	Heating	Cooling	Heating	Cooling
Water Suppr. TE = 30/150	6.41	5.74	7.05	5.39
Water Suppr. TE = 38/182	6.16	5.37	7.00	5.39
No Water Suppr. TE = 38/182	4.64	4.10	5.48	4.47

